# Genome-wide pathway-based quantitative multiple phenotypes analysis

**DOI:** 10.1371/journal.pone.0240910

**Published:** 2020-11-11

**Authors:** Yamin Deng, Shiman Wu, Huifang Fan

**Affiliations:** 1 Statistics Center, First Hospital of Shanxi Medical University, Taiyuan, China; 2 Division of Health Statistics, School of Public Health, Shanxi Medical University, Taiyuan, China; Universita degli Studi di Roma Tor Vergata, ITALY

## Abstract

For complex diseases, genome-wide pathway association studies have become increasingly promising. Currently, however, pathway-based association analysis mainly focus on a single phenotype, which may insufficient to describe the complex diseases and physiological processes. This work proposes a combination model to evaluate the association between a pathway and multiple phenotypes and to reduce the run time based on asymptotic results. For a single phenotype, we propose a semi-supervised maximum kernel-based U-statistics (mSKU) method to assess the pathway-based association analysis. For multiple phenotypes, we propose the fisher combination function with dependent phenotypes (FC) to transform the p-values between the pathway and each marginal phenotype individually to achieve pathway-based multiple phenotypes analysis. With real data from the Alzheimer Disease Neuroimaging Initiative (ADNI) study and Human Liver Cohort (HLC) study, the FC-mSKU method allows us to specify which pathways are specific to a single phenotype or contribute to common genetic constructions of multiple phenotypes. If we only focus on single-phenotype tests, we may miss some findings for etiology studies. Through extensive simulation studies, the FC-mSKU method demonstrates its advantages compared with its counterparts.

## 1. Introduction

Recent years, genome-wide association studies (GWAS) have successfully identified common genetic variants underlying complex diseases. However, traditional GWAS focus on the relationships between single nucleotide polymorphisms (SNPs) and single phenotypes, which can only explain a small portion of heritability [1]. Furthermore, as the stringent significant threshold, gene-based GWAS analysis may miss some moderate effect genes [2]. And the environmental risk factors and interplay of multiple genetic usually together determine the clinical manifestations of complex diseases. Only identifying significant SNPs and genes may be insufficient to describe the pathogenesis of complex diseases [3]. To test the association between a complex disease and a group of candidate genes have become increasingly promising, and may provide more direct biological interpretations of association results [4]. Motivated by completion of the Human Genome Project and the post genome project, researchers proposed pathway-based GWAS study to detect the associations between a complex disease and a group of related genes within a defined biological pathways [5]. Compared with single-SNP based analyses, pathway-based analyses can not only reduce the number of tests by many times but also alleviate multiple comparison problems [6]. Moreover, pathways are highly consistent with populations, which enhance the likelihood of replications. Pathway association studies combine the multiple functionally related genes, which may potentially behave greater power in revealing the biological mechanism within complex diseases [7]. Moreover, genes that may individually have weak effects, but jointly contribute to the diseases risks, will be more likely to be discovered at pathway level than at gene level or at SNPs level. Various pathway-based GWAS methods were developed [2, 7, 8], and have been successfully applied to complex diseases studies.

However, most diseases and physiological processes are complex and comprehensive, and not just a single phenotype variable can be fully described. For example, body mass index (BMI) cannot fully represent the obesity characteristics associated with cardiovascular and cerebrovascular diseases. It only roughly measures the average body weight for a given surface area but cannot express the distribution of fat. Studies have shown that three phenotypes (BMI, waist circumference and hip circumference) can better reflect the risk of cardiovascular and cerebrovascular diseases [9]. Multiple phenotypes can better show underlying molecular mechanisms and provide more information than single phenotype to infer the potentially disease-causing process [10]. Using a single phenotype measurement to define complex diseases is obviously one-sided. And if there is a common genetic predisposition with multiple phenotypes, it will improve the overall signal and further enhance the power of detecting associations. Therefore, GWAS based on multiple phenotypes is more reasonable [3].

For multiple phenotypes analysis, the multivariate analysis of variance (MANOVA) makes an regression of a single SNP on multiple phenotypes, which selects the SNP leading to the smallest p-value (in a gene or pathway) as the gene or pathway level p-value [11, 12]. However, these methods ignore the correlation or interaction between genes in pathways and may lose power when there are multiple weak associations between the genes and multiple phenotypes; some popular methods are to reduce the dimensions of the genetic variants and phenotypes separately to avoid computationally intensive processes. For example, the principal components of heritability (PCH) or principal components analysis (PCA) only choose a few of the most significant association signals among the genetic variants for the association on multiple phenotypes [13, 14]. It is clear that these methods only make use of one or a few components of variants or phenotypes. And another limitation of PCA and PCH is that the final latent variable identified in these models may or may not be related to the phenotypes [15]. Partial least squares method is a regression analysis method to explore the association between multiple dependent variables and multiple independent variables [16]. These methods take into account the correlation degree between the independent and dependent variables, and ensure that the extracted components have strong explanatory power for the dependent variables. However, they fail to give the test statistic distribution of the model parameter, and have to do the permutation test, which has a heavy computational burden for high-dimensional data and cannot be appropriately applied to the ultra-high-dimensional genome-wide level.

Currently, for complex diseases, most pathway-based association studies focus on a single phenotype. It is a natural choice to extend genome-wide pathway-based single phenotype association tests to pathway-based multiple phenotypes analysis. Here, we propose a combination model. In the first part, we introduce a pathway-based method, the semi-supervised maximum kernel-based U-statistic (mSKU) to evaluate the effect (potentially nonlinear) of a pathway on a single phenotype. It has also given strategy to further release the computation burden based on asymptotic results under the setting of high-dimensional. Specially, mSKU considered a semi-supervised testing procedure (a SNP screening step) [17, 18] at the first step, which removes some of the irrelevant SNPs in the pathways. In the second part, which is for a multiple phenotypes analysis, we propose a p-value combination method: the Fisher combination function with dependent phenotypes (FC), which provides an alternative strategy to multiple phenotypes analysis. It transforms the p-values between the gene set and each marginal phenotype individually to test the association between the gene set and multiple phenotypes. We demonstrate the performance of the FC-mSKU method through two real datasets from the Alzheimer Disease Neuroimaging Initiative (ADNI) and the Human Liver Cohort (HLC) study databases. We also provide extensive simulation studies to compare the proposed method with competing methods.

## 2. Statistical methods

### 2.1 The semi-supervised maximum kernel-based U-statistic (mSKU) for the analysis of a single quantitative phenotype

The method is built upon kernel-based test (KBT) [19]. A kernel function is used to identify the similarity between two subjects, which generates the Reproducing Kernel Hilbert Space (RKHS) for the actual function h(∙) to solve linear indivisible situations [20, 21]. It evaluates the joint effect of the pathways to reduce the number of tests and alleviate multiple comparisons problems. A symmetric and positive semidefinite kernel function consists of the (i,j) th element *K*_*ij*_ = *K*(*x*_*i*_,*x*_*j*_) to justify the similarity of the i-th and j-th samples. There are several widely used kernel functions: the linear kernel (k(*x*_*i*_,*x*_*j*_) = *x*_*i*_^*T*^*x*_*j*_+c), Gaussian kernel ($k(x_{i},x_{j})=exp(-\frac{{\left\|x_{i}-x_{j}\right\|}^{2}}{2\sigma^{2}})$) and polynomial kernel (k(*x*_*i*_,*x*_*j*_) = (α*x*_*i*_^*T*^*x*_*j*_+c)^*d*^) functions. If we assume the h (⋅) function denotes the real relationship between a pathway and a phenotype, then different kernel functions generate different “beliefs” about the “real” functions. If the optimal kernel is used, the corresponding KBT test will achieve the maximum power. However, in practice, the optimal h(⋅) function is usually unknown [19]. Here, we introduce a maximum kernel-based U-statistics (mKU) method proposed by Tao et al. [22], which has the power close to the power of the optimal kernel among a vector of M candidate (*K*_1_(∙,∙),*K*_2_(∙,∙),……,*K*_*M*_(∙,∙)) kernel functions.

#### 2.1.1 The maximum kernel-based U-statistics (mKU) with candidate kernel functions for quantitative phenotypes analysis

Tao et al [22] proposed the Kernel-based U-statistic (KU) method with a single kernel function. More details about the KU method please visit the **S1 Appendix** in S1 File. For M candidate (*K*_1_(∙,∙),*K*_2_(∙,∙),……,*K*_*M*_(∙,∙)) kernel functions, they selected the maximum statistic as the final test statistic. Selecting the mKU test statistic is equivalent to the minimum p-value method. However, the minimum p-value method has to bear computational burdens such as permutation to evaluate the statistical significance. Here, the mKU method focuses on the maximum test statistic and takes advantage of the derived asymptotic normality to decrease the calculation pressure under the high-dimensional assumption.

Let n*T*_*n*,*m*_ and $\sigma_{T_{n,m}}^{2}$ be the test statistic and the variance of the m-th kernel function. We define $Q_{m}=\sigma_{T_{n,m}}^{-1}nT_{n,m}$, where *m* = 1,……,*M*. The p‐value is completely determined by *Q*_*m*_, so maximizing *Q*_*m*_ means minimizing a nonlinear function of p-values. The formula for the maximum statistic can be described as follows:

$$Q_{max}=\underset{1\leq m\leq M}{\max}Q_{m}$$ (1)

Definite $\rho_{kl,n}\to \rho_{kl}^{0}$ and *ρ*_*kl*,*n*_ = cov(*Q*_*k*_,*Q*_*l*_) as n→∞ for *k*,*l* = 1,……,*M*. Assuming that each candidate kernel *K*_*m*_ satisfies condition (1), then

$$Q_{max}\overset{d}{\to }\underset{1\leq m\leq M}{\max}Z_{m}$$ (2)

where Z = (*Z*_1_,*Z*_2_,……,*Z*_*M*_)^*T*^ obeys a multivariate normal distribution (The multivariate normal distribution is a generalization of the one-dimensional (univariate) normal distribution to higher dimensions.) with mean *O*_*M*_ and covariance matrix $\Omega^{0}=(\rho_{kl}^{0})$. Through estimating the distribution shapes of statistics, we can not only compute the p-values but also can significantly decrease the run time. And this is also the one of the main contributions of our paper. When the distribution of statistics, the mean and the variance are available, the p-value of the maximum test can be computed as follows:

$$P(Q_{max}>q_{max})=1-P(Q_{max}\leq q_{max})=[1-P(Z\leq q_{max}1_{M})]\{1+o(1)\}$$ (3)

The maximum test strategy has several advantages. First, it achieves the best power among the candidate kernel functions. Second, by taking advantage of asymptotic normality proved in this study, the computational burden is greatly reduced and the results are protected from being overly conservative or inflated. Third, it is suitable for high-dimensional cases and performs well in the calculations. When the number of variants in a pathway is large enough, the proposed method is safe to apply.

#### 2.1.2 The semi-supervised maximum kernel-based U-statistics (mSKU) method for pathway-based analysis

Some researchers considered that without a SNP screening step, using all SNPs to summarize information from a pathway can result in reduced test power for pathway analysis, because of the inclusion of SNPs unrelated to disease [15, 17, 18, 23]. Thus, instead of putting all SNPs in to pathways, we propose a SNP screening step and carries out a within-category selection to identify most important SNPs within each pathway [17]. For each pathway, we suggest these steps:

1. For each SNP, we fit a logistic regression model to compute an association measure with the genotype (0, 1, 2) as the predictor and the disease status as outcome variable.
2. For *i-*th SNP, let *pi* be the p-value of single SNP (i.e. the p-value corresponding to the regression coefficient of logistic model).
3. For a given threshold *α*_*m*_, let Δ_*m*_ = {*SNP*_*i*_∈*G*:*pi*<*α*_*m*_,*i* = 1,…,*n*_*SNP*_} be subset of SNPs below the threshold with association measures. In this paper, we set *α*_*m*_ = 0.05.

### 2.2 The p-value combination method for multiple phenotypes analysis

#### 2.2.1 The Fisher combination function

As performing a univariate association test is much easier than performing a multivariate association test, combining p-values of marginal tests to form joint statistics has become a popular tool to address genetic variants associated with multiple phenotypes [24]. By comparing various combination functions, Birnbaum [25] pointed out that the Fisher combination function was one of the optimal methods [26, 27].

#### 2.2.2 The Fisher combination test with dependent phenotypes

With the Fisher combination method, to test the association between a pathway and multiple phenotypes, we only need to test the association between the pathway and each marginal phenotype individually.

$$T=\sum_{i=1}^{m}-2\;\log\left(p_{i}\right)$$ (4)

where T is defined as a statistic of the Fisher combination function [28], which is used to deduce the association between the pathway and multiple phenotypes. For k correlated multiple phenotypes, we have k marginal p-values: *p*_1_,*p*_2_,……,*p*_*k*_; Under the null hypothesis, Yang et al [29] showed that the distribution of the T statistic is a scale chi-squared distribution ($\alpha ,\;x_{\beta }^{2}$), or equivalently, a gamma distribution (Gamma distribution is related to beta distribution. This distribution arises naturally, and the waiting time between the Poisson distributed events are relevant to each other.) with scale parameter 2α and shape parameter *β*/2. Therefore, if the parameters of α and β are estimated, the global p-value of T can be correspondingly obtained. If we define the mean of T as μ and the variance of T as *σ*^2^, then the corresponding α and β can be computed as α = *σ*^2^/2*μ* and β = 2*μ*^2^/*σ*^2^. The technical details of the derivation of μ and *σ*^2^ are shown in **S2 Appendix** in S1 File.

The global p-value of the T statistic can be calculated by a gamma distribution function:

$$p-value=1-\Gamma (\mu^{2}/\sigma^{2},\sigma^{2}/\mu )$$ (5)

where Γ(*β*/2,2α) is the function of the gamma distribution with scale parameter 2α and shape parameter *β*/2. Thus, we have adjusted k marginal p-values (*p*_1_,*p*_2_,……,*p*_*k*_) to a combined p-value, which is promising way for exploring the relationship between a pathway and the correlated multiple phenotypes.

## 3. Simulation study

We demonstrated the performance of our FC-mSKU method by extensive simulation studies and compared the proposed method with methods of multivariate multiple linear regression (RMMLR), MultiPhen-GATES and O’Brien-VEGAS. Through transforming the genotype and phenotype dataset, RMMLR proposed by Saonli Basu et al. [3] is an improvement on multiple linear regression and achieves a rapid genome-wide association test for multivariate analysis. The R package for the RMMLR method can be downloaded from https://github.com/SAONLIB/RMMLR. Jaeyoon Chung et al. [23] combined two gene-based association tests (GATES and VEGAS) and three multivariate association tests (O’Brien method, TATES, and MultiPhen) to compare the performance (type I error and power) of six multivariate gene-based methods. They concluded that for the studies where individual-level data were available, MultiPhen with GATES can be the best option. When the proportion of missing data are high, it loses substantial power. In this case they suggested O’Brien with VEGAS or TATES with GATES as alternative approaches.

In this study, we applied EpiSIM software [30] to simulate the dataset to mimic the real structures of human pathways. EpiSIM is a simulator that is simple, small but very easy to apply. It offers most general models and allows users to extend their new special models. Thus, all that is needed is to enter the parameters of dataset to analyze and to compile the program, then this software will easily take care of the rest. It allows users to freely set parameters, such as the number of SNPs in pathways, the variation range of MAFs of random SNPs, the model patterns, the linkage disequilibrium, and the main/marginal effects of models that contain one or more SNPs. EpiSIM can be downloaded from https://sourceforge.net/projects/episimsimulator/.

We simulated related quantitative phenotypes with the following model:

$$Y_{ki}=0.02Z_{i1}+0.6Z_{i2}+h(X_{i})+\epsilon_{ki},i=1,\ldots \ldots ,n,k=1,\ldots \ldots ,t$$ (6)

where *ϵ*_*ki*_ (*k* = 1,……,*t*) are random errors of t-dim with a multivariate normal distribution; *Y*_*ki*_ (*k* = 1,……,*t*) are t-dim multiple phenotypes; *Z*_*i*1_~*N*(2,1) and *Z*_*i*2_~*Ber*(0.6) stand for independent covariates; *X*_*i*_ are p-dim SNPs in the pathways. Under each scenario, we simulated 1,000 replicates and set the significance level to 0.05. Under the null hypothesis (i.e., *h*(*X*_*i*_) = 0), we recorded the proportion of results that did not correctly reject the null hypothesis to assess the type I error rates. To assess the power, we set up four scenarios for the *h*(∙) function and recorded the proportion of results that correctly rejected the null hypothesis. Under Scenario M, we supposed h(x) = 0.27(*x*_5_−*x*_10_)+cos(*x*_10_)exp(−*x*_10_^2^/5), where the tenth SNP had nonlinear effects on the five response phenotypes, and the fifth and tenth SNPs had different directions of main effect. Under Scenario N, we presumed h(x) = 0.37*x*_5_+0.62*x*_10_−0.06*x*_15_, where these three SNPs were marginally linearly associated with the multiple phenotypes without inner interaction. Here, to further test the situations when large numbers of SNPs affected the multiple phenotypes, we set the following model:

$$h(x)=b_{P}\sum_{m\in H_{Q}}\alpha_{m}x_{m}+b_{Q}\sum_{mm\prime \in H_{P}}\beta_{mm^{\prime }}x_{m}x_{m^{\prime }}$$ (7)

where *H*_*P*_ was predefined as a set of 60 SNPs with main effects and *H*_*Q*_ was predefined as a set of 90 SNP pairs representing 90 simple interactions. Both {*α*_*m*_,*m*∈*H*_*P*_} and {*β*_*mm*′_,(*m*,*m*′)∈*H*_*Q*_} were independently obtained from a Unif (0, 0.02) distribution, and the parameters were fixed once they were generated for all the simulation replicates. Under Scenario P, we defined the coefficient as (*b*_*P*_, *b*_*Q*_) = (0.07, 2.3), which means a combination of relatively strong interaction influence and a weak main effect. Under Scenario Q, we set up (*b*_*P*_, *b*_*Q*_) = (3.8, 0) to predefine a model with a pure main effect.

**Fig 1:**
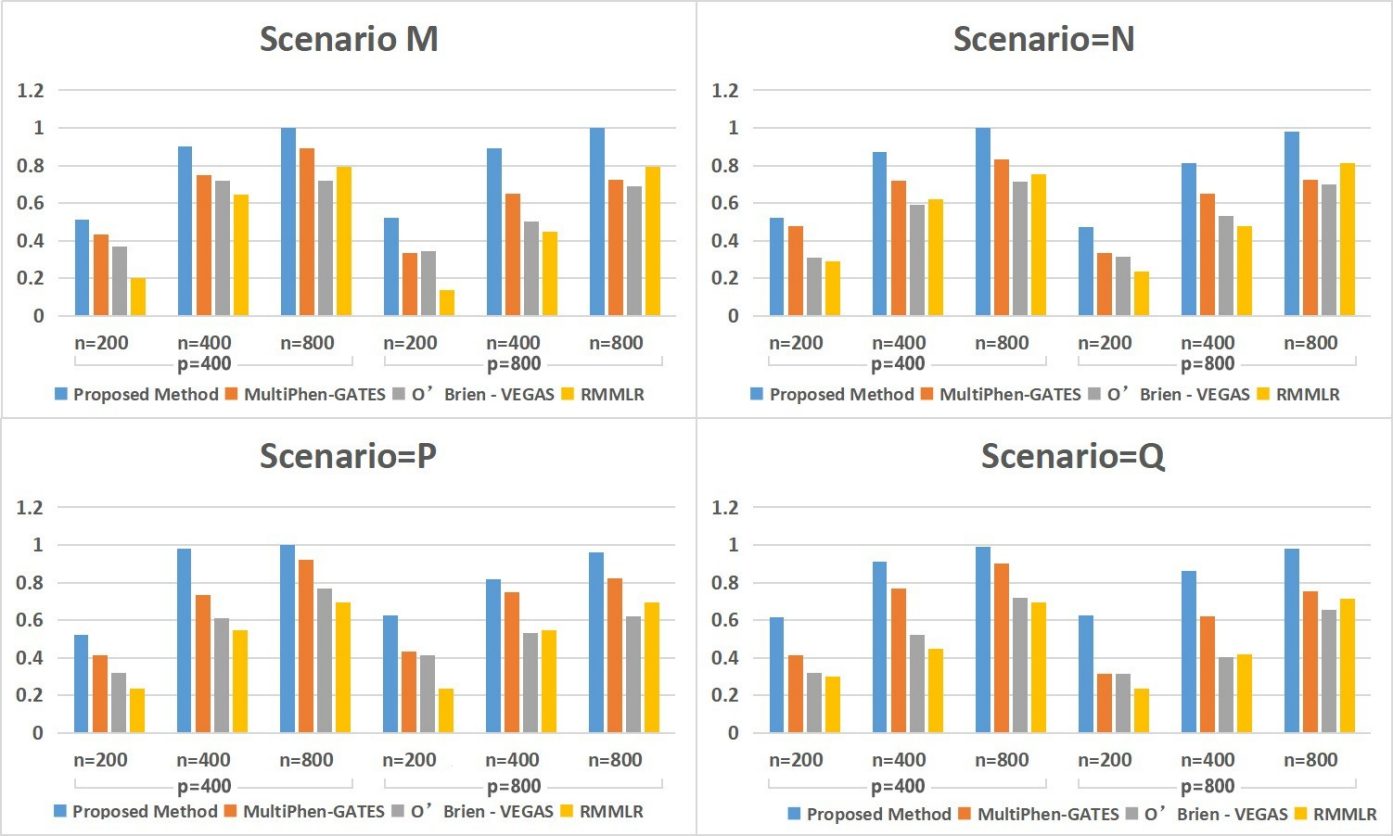
The testing power of the four methods under four scenarios with ρ = 0.2.

Table 1 shows that the method of FC-mSKU, MultiPhen-GATES and RMMLR can maintain reasonable type I error under different settings. However, O’Brien-VEGAS inflated the type I error in some settings. With five related quantitative response phenotypes in Fig 1, as n = 200 increased to n = 800, the FC-mSKU method’s power can reach a satisfactory level. It indicates that through nonparametric kernel-based testing progress, the FC-mSKU method can better capture the potential nonlinear effect of variants (SNPs) within pathways. As the p-dim increased from p = 400 to p = 800, we find that the power of other methods obviously decreases, while the power of the FC-mSKU method can be maintained well. This finding shows that our method has obvious advantages within high-dimensional datasets.

**Table 1:** Under different settings, the experiential type 1 error of four methods.

Data Dimension	Sample Size	Correlation	FC-mSKU	MultiPhen-GATES	O’Brien-VEGAS	RMMLR
*p* = 400	*n* = 200	*ρ* = 0.2	0.051	0.050	0.053	0.057
		*ρ* = 0.7	0.048	0.047	0.061	0.049
	*n* = 400	*ρ* = 0.2	0.050	0.061	0.062	0.041
		*ρ* = 0.7	0.052	0.046	0.071	0.056
	*n* = 800	*ρ* = 0.2	0.049	0.032	0.052	0.063
		*ρ* = 0.7	0.050	0,052	0.073	0.071
*p* = 800	*n* = 200	*ρ* = 0.2	0.046	0.047	0.077	0.063
		*ρ* = 0.7	0.051	0.051	0.063	0.052
	*n* = 400	*ρ* = 0.2	0.047	0.063	0.076	0.047
		*ρ* = 0.7	0.045	0.052	0.061	0.061
	*n* = 800	*ρ* = 0.2	0.047	0.049	0.073	0.072
		*ρ* = 0.7	0.053	0.039	0.080	0.063

**Fig 2:**
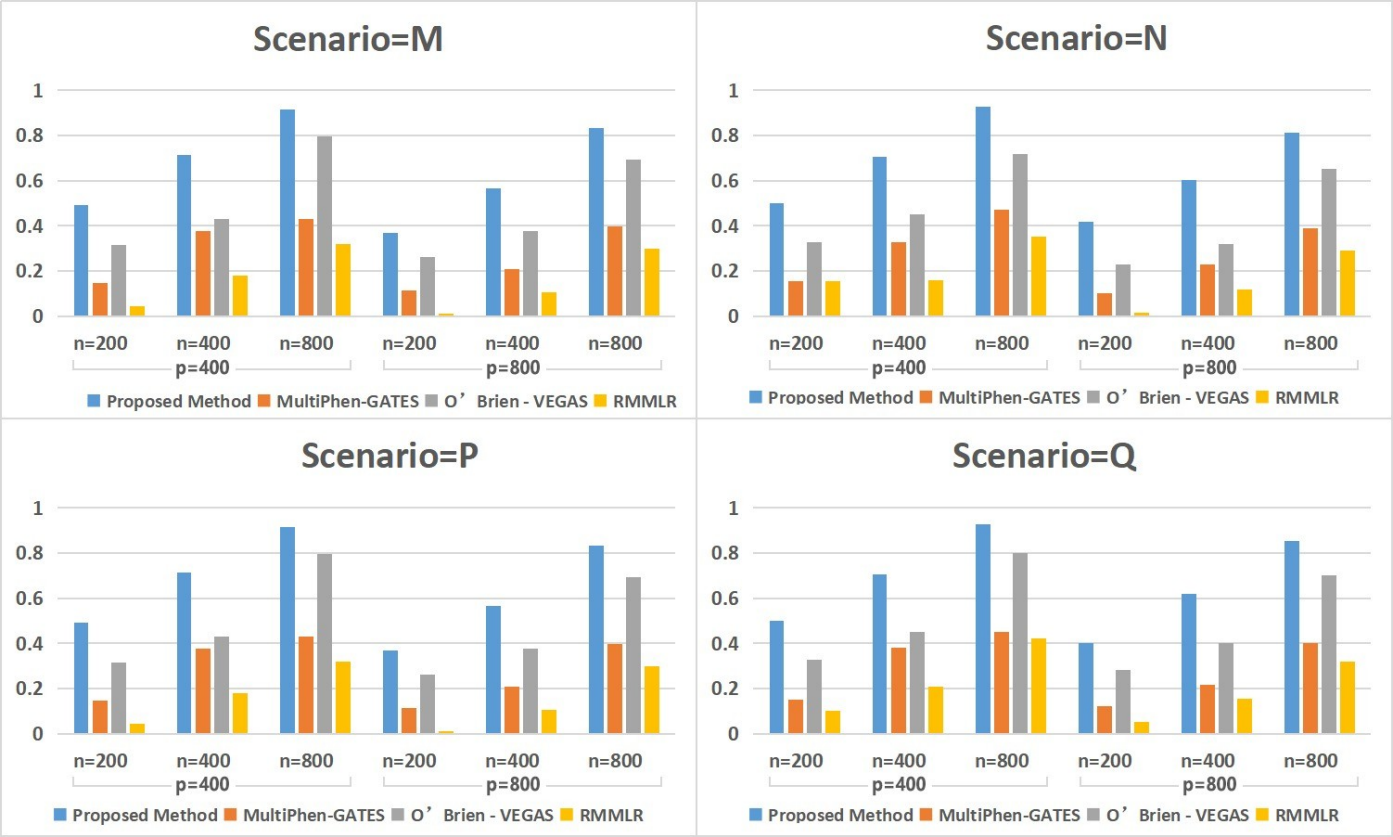
The observed power of the four methods with ρ = 0.7 under four scenarios.

With ρ = 0.7, the testing power of the four methods is shown in Fig 2. Compared with ρ = 0.2, the power of MultiPhen-GATES and RMMLR declines rapidly. On the other hand, O’Brien-VEGAS maintained well and the FC-mSKU method achieves the best performance. This finding indicates that our method can be applicable to phenotypic datasets with strong correlations.

## 4. Real data analysis

### 4.1 Case study Ⅰ: The Alzheimer Disease Neuroimaging Initiative (ADNI) study

One of the most ubiquitous phenomena in natural populations is the variability in numbers. One of the most challenges in population and ecology is to explain and understand the variety and to find the possible underlying variation that may be modified from case-to-case. In this study, we propose the FC-mSKU method to explore the variation of pathways associated with the multiple phenotypes. We applied it to the dataset from the Alzheimer Disease Neuroimaging Initiative (ADNI) study. Data used in preparing this article were obtained from the Alzheimer's Disease Neuroimaging Initiative database (http://www.loni.ucla.edu/ADNI).Study resources and data from the North American ADNI study are available through this website, including Alzheimer’s disease patients, mild cognitive impairment subjects, and elderly controls. Alzheimer Disease (AD), which is caused by brain tissue deterioration, is an irreversible neurodegenerative disease. AD affects over approximately 5.5 million Americans, especially among people over the age of 65, and there are no prevention methods, and no cures have been discovered. Aiming at the early detection of AD and tracking its development, the ADNI is a longitudinal multicenter study to develop clinical, imaging, biochemical biomarkers and genetic analysis of AD.

ADNI provides pre-calculated volumes of five cortical regions (entorhinal cortex, ventricles, hippocampus, fusiform gyrus and middle temporal gyrus) and the whole brain. In total, 490 samples were screened from the ADNI1 and ADNI2 studies, and we only selected the samples with both complete phenotypic information for the volumes of brain cortical regions and complete genetic information. We excluded the SNPs with low MAFs (minor allele frequencies) <0.05 or failed to pass through the Hardy-Weinberg exact test. The total number of SNPs eventually included in the research was 620,901. And they were further mapped into 315 pathways through the KEGG (Kyoto Encyclopedia of Genes and Genomes) by the clusterProfiler R package. On the one hand, it is well known that the volume of the whole brain is significantly reduced in patients with AD [31]. On the other hand, the volumes of these five brain regions are also reduced to varying degrees. Therefore, we first selected the five cortical regions as multiple phenotypes, which can better describe the pathological changes in the brains of AD patients. Then, we compared the test results with only taking the whole brain volume as a single-phenotype response variable. We log-transformed the five phenotypes as the response variables and took age, sex, education level and whether patient had the APOE4 gene as the covariates.

**Fig 3:**
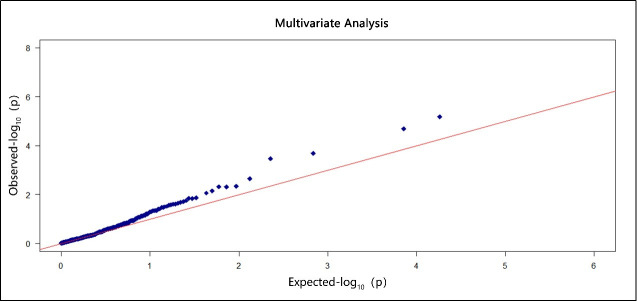
The Q-Q-plot of p-values shows the results of multivariate analysis. The x-axis denotes the expected p-value (-log 10), while the y-axis shows the observed p-value (-log 10). The red diagonal line has slope 1 and intercept 0.

For each cortical region, we performed a pathway-based genome-wide association analysis with the mSKU method. The Q-Q plots of p-values for the five individual phenotypes are shown in **S1 Fig** in S1 File. As the five cortical regions can more specifically and accurately describe brain changes in AD patients than the single whole brain, we regarded the five cortical regions as multiple phenotypes and applied the proposed FC method for the multiple phenotypes analysis. The Q-Q plot is shown in Fig 3. Based on the results of single-phenotype tests, we identified three pathways (hsa05216, hsa01200, hsa00533) associated with the entorhinal cortex, two pathways (hsa05211 and hsa04211) associated with the ventricles, three pathways (hsa00410, hsa04972 and hsa03022) associated with the hippocampus, and three pathways (hsa00533, hsa04120 and hsa04137) associated with fusiform and middle temporal gyri. Then, when we extracted the five cortical regions as a multiple phenotypes response variable, four pathways (hsa00533, hsa04137, hsa05216 and hsa05010) were identified. On the other hand, when we chose the whole brain as a response variable in the single-phenotype test, we only discovered a single pathway (hsa04144). Thus, if the goal is to identify the pathways associated with changes in AD brain volume, the proposed FC-mSKU method is a better choice.

### 4.2 Case study Ⅱ: The Human Liver Cohort (HLC) study

We applied the FC-mSKU method to an analysis of the Human Liver Cohort (HLC) study to explore the genetic architecture of the P450 enzyme in humans. The database can be downloaded from the Sage Bionetworks platform with the Synapse ID of syn4499 (https://www.synapse.org/#!Synapse:syn4499). We chose the activity measurements of the P450 enzyme as the phenotypes of interest [26]. We extracted 170 unrelated Caucasian individuals from three independent tissue collection centers. SNPs with a minor allele frequency of less than 5% or genotyping rate of less than 90% were removed, leaving 312,082 SNPs. And then they were mapped into 312 pathways defined by the KEGG, and the annotation process was based on the clusterProfiler R package. The HLC study contains a total of nine enzyme activity measures that belong to three families: CYP1, CYP2, and CYP3, which probably decompose 95% of drugs used in clinical practice [32]. According to the correlation coefficient, we selected six enzymes (CYP1A2, CYP3A4T, CYP2C8, CYP2B6, CYP2C9 and CYP2A6) as the response variables and used age and sex as covariates. We chose to log-transform the six phenotypes as the response variables to normalize each phenotype. Pearson correlation coefficients of the six phenotypes show a moderate correlation between them ranging from 0.34 to 0.51 (see **S2 Fig** in S1 File). Thus, we hypothesize that there may common pathways associated with multiple p450 enzymes.

**Fig 4:**
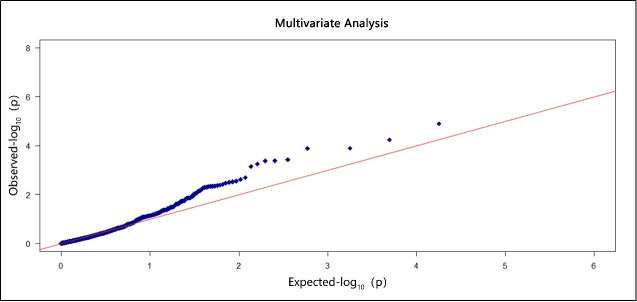
It shows the Q-Q-plot of p-values by FC to test the association between pathways and multiple phenotypes. The x-axis denotes expected-log 10 (p-value), while the y-axis shows observed-log 10 (p-value). The red diagonal line has slope 1 and intercept 0.

For each phenotype, the estimated p-values by the proposed mSKU method are summarized via the Q-Q plots in **S3 Fig** in S1 File. As the six marginal variables are correlated and have similar biological functions, we regarded the six phenotypes as multiple phenotypes and applied the Fisher combination test with dependent phenotypes (FC) to explore the common pathways associated with them. The Q-Q plot of the multivariate analysis is shown in Fig 4. According to the results of the single-phenotype tests, the numbers of pathways identified to be associated with individual phenotypes were as follows: one pathway (hsa00982) for CYP2A6, two pathways (hsa04015, hsa00982) for CYP2B6, three pathways (hsa00980, hsa00982 and hsa00983) for CYP1A2, two pathways (hsa04910 and hsa05100) for CYP3A4T, one pathway (hsa04140) for CYP2C8, and 0 pathway for CYP2C9. On the other hand, three pathways (hsa00983, hsa00982 and hsa04950) were identified to be associated with multiple phenotypes. Among them, hsa00983 and hsa00982 were also identified by the single-phenotype test. Specifically, hsa04950 was newly discovered. This study implies that if we ignore the association among the six phenotypes and only conduct a single-phenotype test, we might miss something interesting.

## 5. Discussion

As the biological functions of pathways are more directly consistent with human populations, genome-wide pathway-based for complex diseases may not only provide more chances to reveal functional mechanisms, but also enhance the likelihood of replication. Moreover, for complex diseases, genome-wide association analysis with multiple phenotypes is more reasonable. For multiple phenotypes genome-wide association analysis in gene-set- or pathway-based analyses, the proposed FC-mSKU method is suggested to be a promising strategy. It proposes efficient measurements to reduce the computational burden, which has expanded its practical applicability. It has the following advantages: (1) the FC-mSKU method is powerful in the association analysis of ultrahigh dimensional SNPs and multiple phenotypes, and it also maintains reasonable type I error. Furthermore, through the application of the asymptotic results of the model statistics, the proposed method has significantly decreased the computational burden. (2) The kernel-based mSKU method jointly evaluates a subset variants in pathways and captures the potentially complex interaction mechanism among the gene set. It obviously performs better than many models that are restricted to linear interactions. (3) For multiple phenotypes analyses, the improved Fisher combination function with dependent variables not only considers the correlation between multiple phenotypes but also improves the computational speed.

With the ADNI study, we identified four pathways (hsa00533, hsa04137, hsa05216 and hsa05010) associated with the multiple phenotypes of brain atrophy in AD patients. Hsa05010, whose pathway name is Alzheimer Disease, has been proven to be closely associated with AD in the literature [33, 34]; However, hsa05010 was only identified by the multiple phenotypes-based analysis in this study but was not identified in single-phenotype tests. Hsa05216 has been proven by the Crosstalk of Dysfunctional Pathways in Alzheimer Disease Brains [35]. With the HLC study, we identified three pathways (hsa00983, hsa00982 and hsa04950) associated with the multiple phenotypes of the p450 enzyme. Hsa00982 is the pathway of “Drug metabolism—cytochrome P450”. Hsa00983 is the pathway of ‘‘drug metabolism-other enzymes”, which is responsible for the processing drug Metabolism that are involved in the inhibition of DNA replication, such as azathioprine and fluorouracil [36, 37].

From the analyses of the HLC study and ADNI study, the FC-mSKU method allows us to specify which pathways are specific to a particular phenotype or contribute to the common genetic construction. It is known that pathways usually do not work in isolation, and cellular pathways with complex molecular networks are often more directly involved in the diseases. Therefore, if we only focus on the single-phenotype test, we may miss some ideas for etiology research. However, the proposed method only analyzed continuous multiple phenotypes. In the literature, many outcomes of complex diseases are measured by both discrete and continuous scales. For example, dizziness (binary outcome) and blood pressure (continuous outcome) are both very important phenotypes in hypertension research. In future studies, we will focus on mixed multiple phenotypes analysis (binary and continuous) in genetic association studies, which may be the most common judging criteria of complex diseases.

## Supporting information

S1 File (DOCX)
