# Supplementary material for: Genome-wide pathway-based quantitative multiple phenotypes analysis
Source: PLoS One. 2020 Nov 11;15(11):e0240910. doi: 10.1371/journal.pone.0240910 (PMC7657528; doi:10.1371/journal.pone.0240910)
Supplement: S1 File — (DOCX) [file pone.0240910.s001.docx]

**Supplemental file for “*Genome-wide pathway-based quantitative multiple phenotypes analysis*”**

Yamin Deng^1,2*^ , Shiman Wu^1^ , Huifang Fan^1^

^1^ Statistics Center, First Hospital of Shanxi Medical University

^2^ Division of Health Statistics, School of Public Health, Shanxi Medical University

* Corresponding author:

E-mail: [yamin.deng@sxmu.edu.cn](mailto:yamin.deng@sxmu.edu.cn) (DYM)

**S1 APPENDIX**

**Kernel-based U-statistic (KU) method with a single kernel function.**

To model the association between a pathway and a quantitative phenotype, the following semiparametric regression model is considered:

$Y_{i}=\alpha^{T}W_{i}+h\left( x_{i} \right)+\varepsilon, i=1,2,\ldots\ldots,n$ (1)

where $n \left( i=1,2,\ldots\ldots,n \right)$ represent independent samples observed in the study design. For the *i-*th sample, $Y_{i}$ is the quantitative response variable, $x_{i}={(x_{i1},\ldots\ldots x_{ip})}^{T}$is a vector of p-dim variants in the pathway, $W_{i}={(W_{i1},W_{i2},\ldots\ldots W_{iH})}^{H}$is a vector of H-dim covariates, $h\left( \cdot\right)$is an unknown function and $\varepsilon_{i}$is the random error. Here, there are two hypotheses: $H_{0}:h\left( \cdot\right)=0$ and $H_{1}:h\left( \cdot\right)\neq0$.

The following model is considered:

$T_{n}={\frac{1}{n\left( n-1 \right)}\sum_{i\neq j} K\left( X_{i},X_{j} \right)\left( Y_{i}-\hat{Y}_{i} \right)\left( Y_{j}-\hat{Y}_{j} \right)}/{\hat{\sigma}^{2}}$ (2)

where$\hat{Y}$and$\hat{\sigma}^{2}$are parameters estimated under the null model$: Y_{i}=\mu+\alpha^{T}W_{i}+\epsilon_{i}$. Let$\tilde{W}_{n\times(L+1)}=[1_{n},W_{n\times L}]$and$A=\tilde{W}{(\tilde{W}^{T}\tilde{W})}^{-1}\tilde{W}^{T}$; then, we can obtain ${{\hat{\sigma}^{2}=Y}^{T}(I-A)Y}/\left( n-L-1 \right)$ and $\hat{Y}=AY$. $V_{k}=\sum_{m=1}^{\infty} \lambda_{m}^{k}$ is defined for any positive integer $k$.

If ${V_{4}}/{V_{2}^{2}}\to0$ as $p\to\infty$ holds (3)

Under the null hypothesis of no significant genetic effect ($h\left( \cdot\right)=0$), the$T_{n}$follows asymptotic normality:

$\sigma_{T_{n}}^{-1}nT_{n}\underset{\to}{d}N(0,1$) (4)

where $\sigma_{T_{n}}^{2}$ can be estimated by the following formula:

$\hat{\sigma}_{T_{n}}^{2}=\frac{1}{n^{2}}\{\left( 2-\frac{12}{n^{2}}+\frac{6\hat{\Delta}}{n} \right)tr\left( B^{2} \right)-\left( \frac{2}{n}+\frac{\hat{\Delta}}{n} \right){tr}^{2}\left( B \right)+\hat{\Delta}tr(B\circ B)\}$ (5)

where $B=HK_{n}^{O}H; H=I-A$;$\circ$ is the elementwise product; $\hat{\Delta}=n^{-1}\sum_{i=1}^{n} {\left[ {(Y}_{i}-\hat{Y_{i}} \right)/\hat{\sigma}]}^{4}$-3; then, the p-value under the null hypothesis can be obtained:

$p-value=1-\emptyset(\sigma_{T_{n}}^{-1}nT_{n})$ (6)

where $\emptyset(\cdot)$ is the cumulative density function of the standard normal distribution.

**S2 APPENDIX**

**The technical details of the derivation of** $\boldsymbol{\mu}$ **and** $\boldsymbol{\sigma}^{\boldsymbol{2}}$ **of Fisher combination test with dependent phenotypes.**

Assuming the test statistic for the i-th phenotype with $Z_{i}$ (i=1, 2,….., m), the corresponding p-value can be defined as $p_{i}=2\emptyset(-\left| z_{i} \right|)$, where $\emptyset$ is a standard Gaussian distribution function. Under the null hypothesis, the distribution of T can be approximated by a Gaussian distribution with mean $\mu=E\left[ T \right]=2m$and variance defined as:

$$\sigma^{2} =Var\left[ T \right]$$

$= Var\{\sum_{i=1}^{m} -2\log\left( p_{i} \right) \}$

$=$ $\sum_{i=1}^{m} Var\left\{ -2\log\left( p_{i} \right) \right\}+\sum_{i\neq j} cov\{-2\log\left( p_{i} \right),-2\log\left( p_{j} \right)\}$

$=4m+\sum_{i\neq j} cov\{-2\log\left( p_{i} \right),-2\log\left( p_{j} \right)\}$ (7)

Furthermore, before calculating the variance of$T,$we can first compute the covariance of each pair$\left( i,j \right)$as:

$$cov\{-2\log\left( p_{i} \right),-2\log\left( p_{j} \right)\}$$

$=E\left\{ \left[ -2\log\left( p_{i} \right) \right]\left[ -2\log\left( p_{j} \right) \right] \right\}-E\{-2\log\left( p_{i} \right)\}E\{-2\log\left( p_{j} \right)\}$

$=4\int_{-\infty}^{\infty} \int_{-\infty}^{\infty} log\{2\emptyset(-\left| Z_{i} \right|)\}\log\left\{ 2\emptyset\left( -\left| Z_{j} \right| \right) \right\}dF\left( Z_{i},Z_{j} \right)-4$ (8)

Let $\delta_{ij}=cov\{-2\log\left( p_{i} \right),-2\log\left( p_{j} \right)\}$. Thus, $\delta_{i,j}$ is a function that denotes the correlation$\rho_{i,j}$ between $Z_{i}$ and $Z_{j}$. The relationship between $\delta_{i,j}$ and $\rho_{i,j}$ can be estimated as a tenth-order polynomial:

$\delta_{ij}=c_{1}\rho_{i,j}^{2}+c_{2}\rho_{i,j}^{4}+c_{3}\rho_{i,j}^{6}+c_{4}\rho_{i,j}^{8}+c_{5}\rho_{i,j}^{10}$ (9)

Two steps are taken to accurately estimate $\delta_{i,j}$and remove potential bias. First, since sample correlation $\hat{\rho}_{i,j}$ is a bias estimator of $\rho_{i,j}$, $\rho_{i,j}$ can be appraised by the bias-corrected sample correlation $\hat{\gamma}_{i,j}$:

$\hat{\gamma}_{i,j}= \hat{\rho}_{i,j}(1+\frac{1-\hat{\rho}_{i,j}^{2}}{2(n-3)})$ (10)

where $n$ is the sample size. Then, $\delta_{i,j}$ can be defined as:

$\delta_{ij}=f\left( r \right)=c_{1}\gamma^{2}+c_{2}\gamma^{4}+c_{3}\gamma^{6}+c_{4}\gamma^{8}+c_{5}\gamma^{10}$ (11)

Second, further bias can be estimated by:

$\frac{c_{1}}{n}{(1-\hat{\gamma}_{i,j}^{2})}^{2}$ (12)

Therefore, the unbiased estimator of $\delta_{i,j}$ is ${f\left( \hat{\gamma}_{i,j} \right)-\frac{c_{1}}{n}(1-\hat{\gamma}_{i,j}^{2})}^{2}$, and the unbiased estimator of $\sigma^{2}$ is $\sigma^{2}=Var\left( T \right)=4 m+\sum_{i\neq j} {(f\left( \hat{\gamma}_{i,j} \right)-\frac{c_{1}}{n}(1-\hat{\gamma}_{i,j}^{2})}^{2})$.

**S1 Fig** shows the QQ plots of the five traits with the proposed pathway-based single trait analyses. There is no indication of p-value inflation.


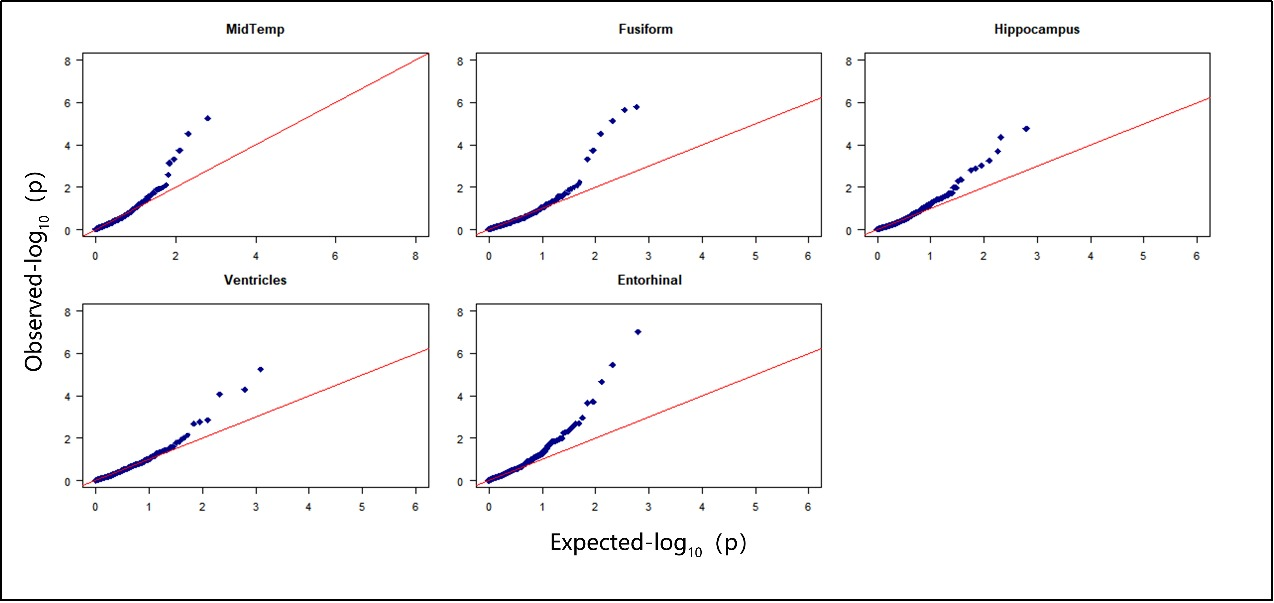


S1 Fig. The Q–Q plots of the p-values show the results of marginal association tests between the pathways and each variable. The x-axis denote expected p-value (−log 10), while the y-axis denote the observed p-value (−log 10). The red diagonal lines have slope 1 and intercept 0.

**S2 Fig** shows the correlation between the six p450 enzymes with the Pearson correlation coefficient ranging from 0.34 to 0.51.


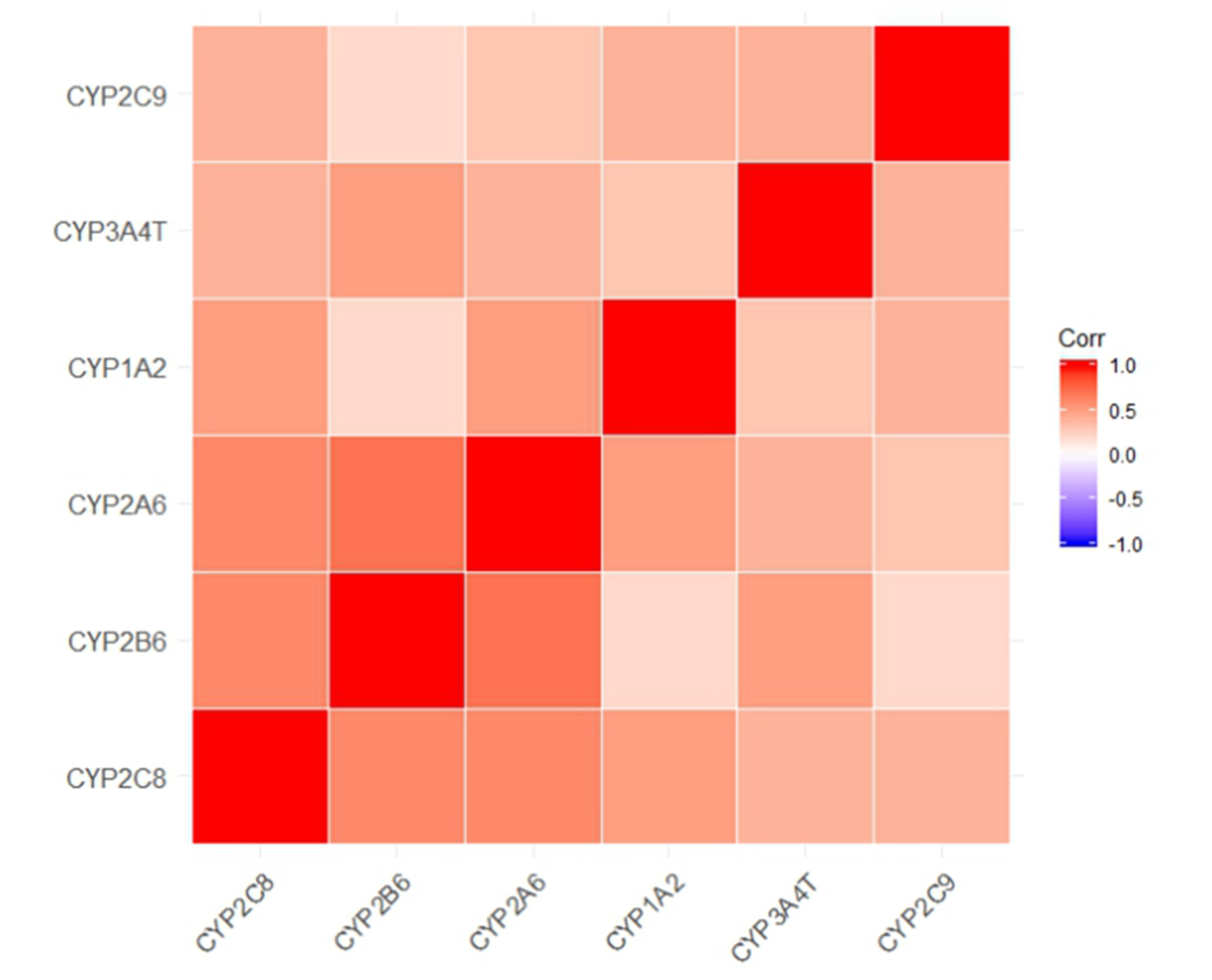


S2 Fig. The correlation coefficients of the six phenotypes for the p450 enzymes (CYP1A2, CYP3A4T, CYP2C8, CYP2B6, CYP2C9 and CYP2A6).

**S3 Fig** shows the QQ plot of the six p450 enzymes with the proposed pathway-based single trait analyses. Again, there is no sign of p-value inflation.


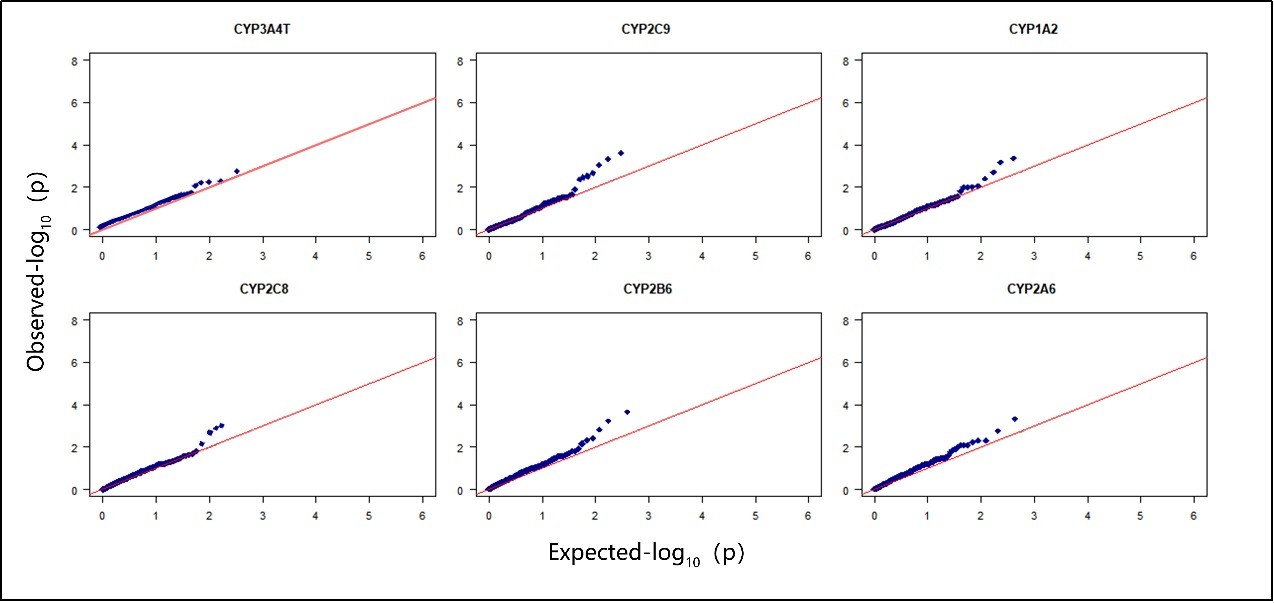


S3 Fig. The Q-Q plots of the p-values show the results of marginal association tests between the pathways and each variable. The x-axis denote the expected p-value (-log 10), while the y-axis denote the observed p-value (-log 10). The red diagonal lines have slope 1 and intercept 0.
